# Case Report: Bevacizumab-induced renal-limited TMA and FSGS-like lesions in a kidney transplant recipient

**DOI:** 10.3389/fonc.2025.1638274

**Published:** 2025-10-06

**Authors:** Xingxing Jian, Ge Liu, Shuangyan Yu, Jie Sheng

**Affiliations:** Department of Nephrology, The Second Affiliated Hospital of Dalian Medical University, Dalian, Liao Ning, China

**Keywords:** bevacizumab, anti-VEGF drugs, renal-limited TMA, kidney transplantation, tumor

## Abstract

Bevacizumab, an anti-VEGF agent commonly employed in cancer therapy, can induce renal vascular endothelial and podocyte damage by inhibiting VEGF, leading to renal disease. We present a case of nephrotic syndrome and renal insufficiency arising from small intestinal mucinous adenocarcinoma 17 years post-renal transplantation, treated with bevacizumab and sindilizumab. Renal biopsy confirmed renal-limited thrombotic microangiopathy (TMA) with FSGS-like lesions in the transplanted kidney, attributed to bevacizumab. Upon discontinuation of the antitumor drugs and initiation of treatment with telmisartan, fenoldopam, and ambrisentan, proteinuria markedly decreased and eGFR improved. No tumor recurrence was observed over a follow-up period exceeding six months.

## Introduction

Renal transplant recipients exhibit a significantly higher incidence of new tumors compared to the general population. With the increase in long-term survival rates among these recipients, the occurrence of concurrent tumors during follow-up has also risen (1). Bevacizumab, an anti-VEGF drug commonly used in treating advanced tumors, poses a nephrotoxicity risk due to its mechanism of action. VEGF is crucial for maintaining glomerular endothelial cell homeostasis and podocyte function. It is secreted by podocytes and modulates endothelial pore structure and antithrombotic ability via VEGFR-2 (2). Bevacizumab induces glomerular endothelial cell damage by inhibiting VEGF-A’s interaction with its receptor (3). Pathological analysis reveals that 50%-73% of such renal injuries manifest as thrombotic microangiopathy (TMA), while 27% exhibit focal segmental glomerulosclerosis (FSGS)-like lesions (4, 5). Clinical evidence suggests that the incidence of bevacizumab-induced proteinuria in patients with solid tumors varies between 21% and 63%, although the occurrence of nephrotic-range proteinuria associated with bevacizumab remains relatively uncommon (6). Due to immunosuppression and the susceptibility of endothelial cells in transplanted kidneys, recipients of kidney transplants are at an elevated risk for such complications: among more than 10 reported cases, 8 developed severe proteinuria, and 6 were pathologically confirmed to have TMA. However, no biopsy-confirmed cases of concurrent FSGS-like lesions have been documented (7, 8).

This article presents a case of renal-limited thrombotic microangiopathy (TMA) with FSGS-like lesions post-renal transplantation, complicated by mucinous adenocarcinoma of the small intestine and bevacizumab treatment. It underscores the importance of prompt renal biopsy in transplant patients with tumor-related renal injury during antitumor therapy to determine the underlying cause. Multidisciplinary collaboration is essential for personalized treatment.

## Case presentation

The patient, a 62-year-old male, presented with a history of double lower limb edema 22 years ago. At that time, his blood creatinine level was >700μmol/L, and urine protein was 2+, with no hematuria. No renal biopsy was performed. After one year of hemodialysis treatment, the patient underwent kidney transplantation. Following the transplant, he was prescribed tacrolimus and mycophenolate mofetil for anti-rejection therapy, and his renal function remained normal, with no proteinuria or hematuria. Four years ago, the patient experienced abdominal pain. Abdominal CT imaging revealed localized thickening and aneurysmal dilatation of the left ileum wall, with the thickest point measuring approximately 2.3 cm. Multiple irregularly shaped nodular shadows were observed in the abdominal cavity, including a large one measuring around 5.2cm×4.3 cm. Additionally, a rounded hypoplastic shadow was noted on the right side of the pelvis, measuring approximately 6.0 cm×4.4 cm. Abdominal exploration was conducted, and multiple masses were identified in the omentum, mesentery, and pelvis. Two large omental nodules were excised, and the postoperative pathological diagnosis was mucinous adenocarcinoma. Immunohistochemistry analysis revealed the following: CDX-2 (+), SATB2 (–), PAX8 (–), MSH2 (+), MSH6 (+), PMS2 (+), MLH1 (+), CK20 (+), and C-erbB-2 (0). Molecular pathology testing showed a wild-type BRAFV600E. The clinical diagnosis was mucinous adenocarcinoma of the small intestine, classified as TxN+M1 stage IV with multiple metastases in the abdominopelvic cavity.

**Figure 1:**
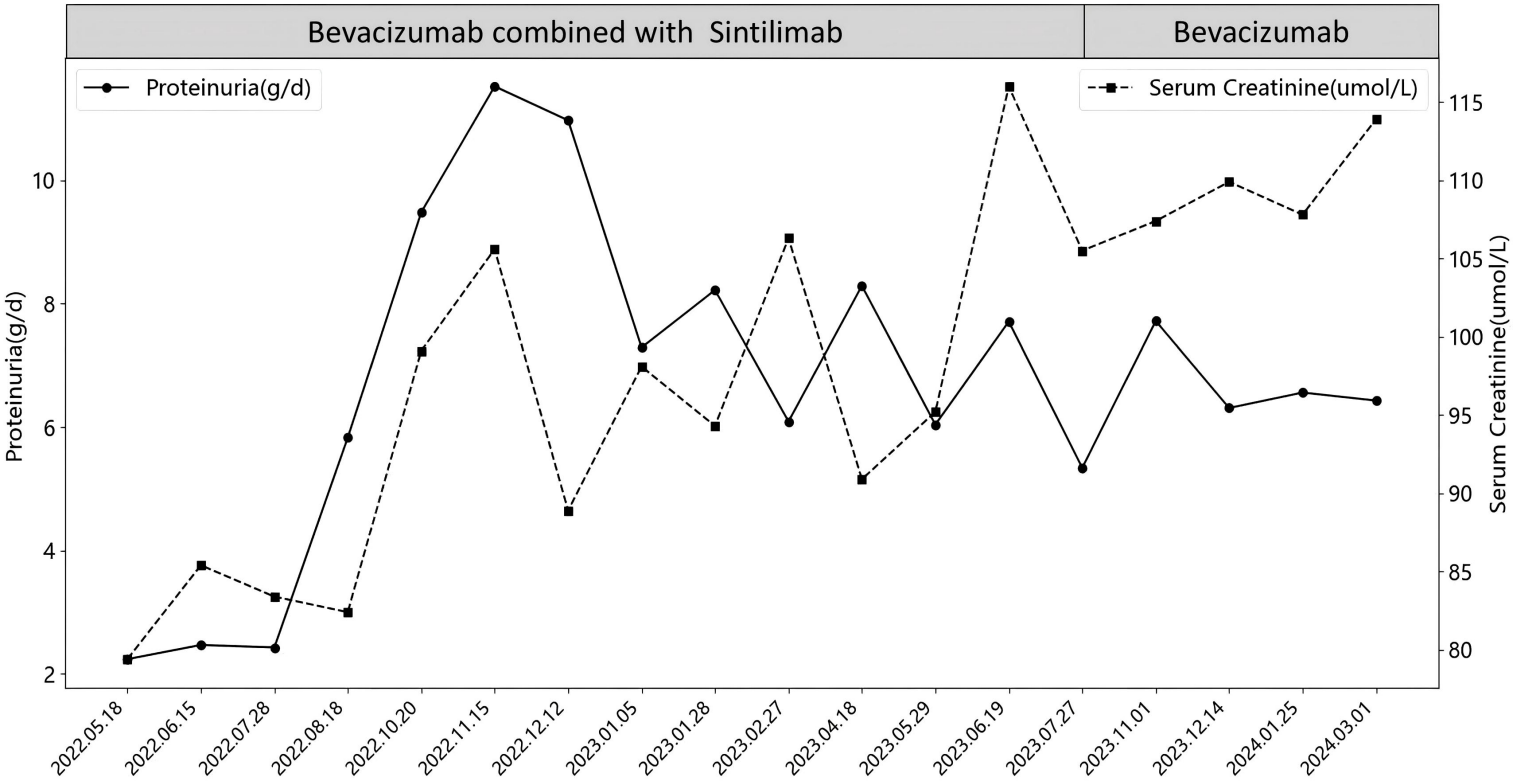
Changes in proteinuria and serum creatinine during anti-tumor treatment.

The anti-tumor treatment regimen consisted of bevacizumab (500–700 mg ivgtt Q21d) and sindilizumab (200 mg ivgtt Q21d), with renal function and urine monitoring conducted each cycle. Initially, the patient exhibited no proteinuria, and blood creatinine levels ranged from 60-87μmol/L. After 15 months(2022.05.18), proteinuria and elevated blood creatinine emerged, with 24-hour urinary protein increasing from 2.24 g/d to 11.52 g/d and creatinine peaking at 116μmol/L. Following evaluation by oncologists and nephrologists, sintilimab was discontinued, and bevacizumab monotherapy continued for 7 months. However, proteinuria persisted, with 24-hour urinary protein between 4.72-6.56 g/d and creatinine levels fluctuating from 106-119μmol/L. Ultimately, bevacizumab was also discontinued, as detailed in **Figure 1**.

After discontinuing all antineoplastic medications for 2 months, the patient was admitted to our department for further management, presenting with a 24-hour urine protein quantification of 7.79 g/d and a serum creatinine of 133μmol/L. The patient had a 10-year history of hypertension, managed with regular oral chlorosartan and benidipine, with blood pressure fluctuating between 130-170/80-90mmHg.Laboratory investigations revealed a 24-hour urine protein of 7.93 g/d, serum albumin of 25.65 g/L, serum creatinine of 143μmol/L (estimated glomerular filtration rate of 44.9 mL/1.73m^2^), hemoglobin of 140 g/L, platelet count of 141×10^9^/L, and lactate dehydrogenase (LDH) of 187.01U/L. Complement C3 was 0.78g/L, and the patient tested negative for anti-phospholipase A2 receptor (PLA2R) antibody, vasculitis antibody, immunoglobulin, anti-double-stranded DNA antibody, hepatitis B surface antigen, BK virus, and cytomegalovirus. The panel for panel-reactive antibody was also negative. Imaging studies showed a transplanted kidney measuring 12.7×6.8×6.9 cm, with clear corticomedullary differentiation and good blood flow perfusion. Abdominal and pelvic CT revealed no bowel wall thickening, no abnormal lesions in the abdominal cavity, no enlarged lymph nodes in the retroperitoneum and abdominopelvic cavity, and a rounded low-density focus on the right side of the pelvis, measuring approximately 1.8×1.5 cm.

**Figure 2:**
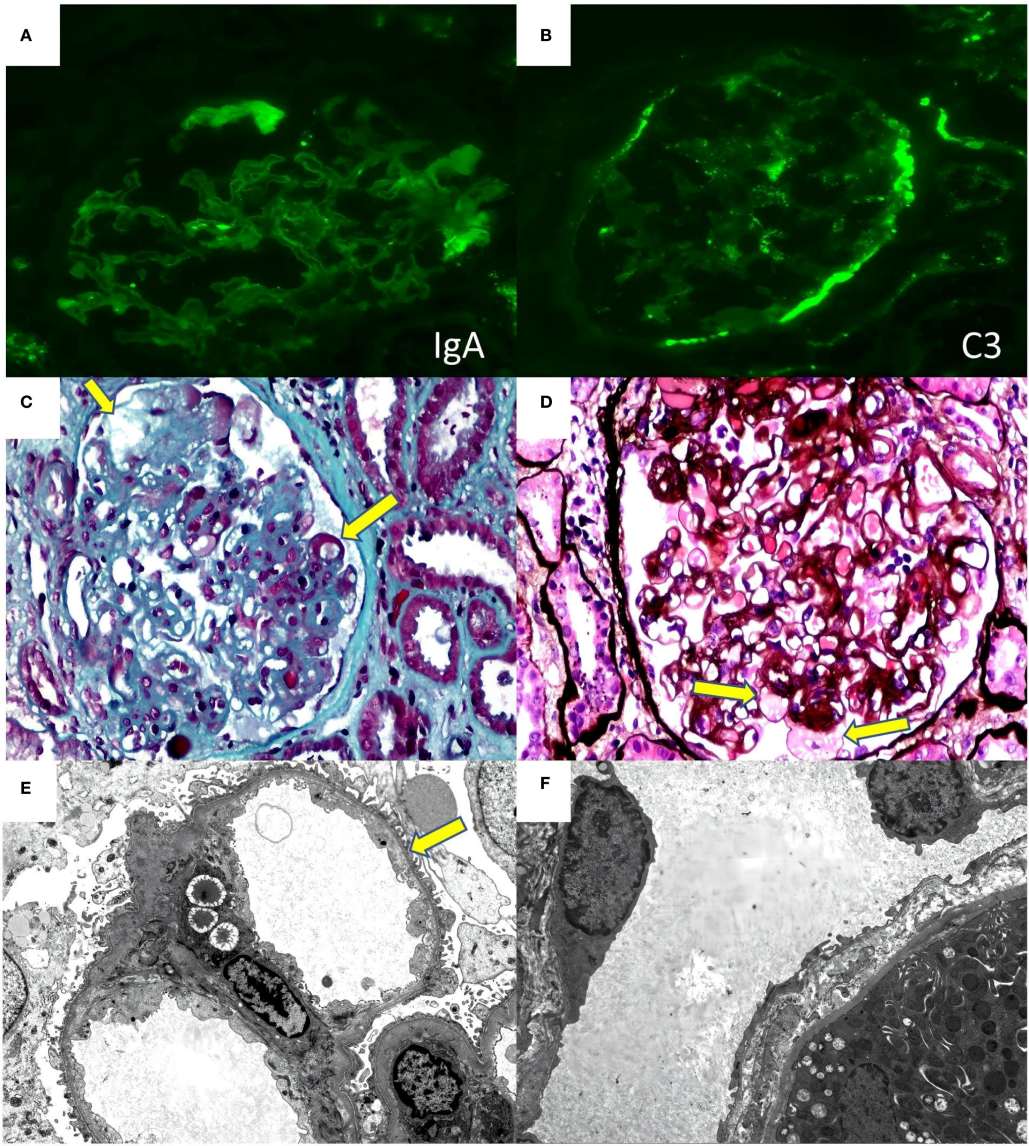
Renal biopsy findings of the case **(A)** immunofluorescence for IgA. **(B)** immunofluorescence for C3. **(C)** (masson 40×10) mesangiolysis、subendothelial space widening (yellow arrowhead) **(D)** (PASM+HE 40×10)Aneurysmal Dilatation (yellow arrowhead) **(E)** (electron microscopy 2000×)subendothelial space widening (yellow arrowhead) **(F)** (electron microscopy 2000×) There is no diagnostically significant stratification of the peritubular capillary basement membrane.

Renal Biopsy Findings: Immunofluorescence revealed IgA-like thread-like deposits in capillary collaterals (**Figure 2A**). IgM (2+) and C3 (2+) showed diffuse, globular, and granular deposits in tethered areas and some capillary collaterals. Positive C3 was observed on the wall of Bowman’s capsule (**Figure 2B**). Exudative-like changes were noted in IgM, C1q, and C4 segments, while IgG, its subclasses (IgG1, IgG2, IgG3, IgG4), PLA2R, and light chains Kappa and Lambda were negative. No deposition of immunoglobulins or complement components was found outside the glomerulus. C4d staining was negative. Immunohistochemical staining for SV40 was also negative. The biopsy sample contained 27glomeruli on light microscopy: global glomerular sclerosis in five glomeruli, segmental sclerosis in fourteen other glomeruli, resulting in a sclerosis rate of 70.37%. The glomerular capillary loops remained patent, with most peripheral collaterals displaying stratification, mesangiolysis, aneurysmal dilatation, and hyaline degeneration (**Figures 2C, D**). Notably, podocyte proliferation was observed in 2 glomeruli (7.4%). The tubulointerstitium exhibits mild chronic lesions (15%), characterized by focal tubular atrophy, basement membrane thickening, sporadic shedding of the brush border of tubular epithelial cells, diffuse cloudy swelling, granular degeneration, and vacuolar degeneration of tubular epithelial cells. A small amount of protein casts is present in the tubular lumen, and no tubulitis is noted. There was mild edema in the renal interstitial region, with small amount of inflammatory cells infiltration, and mild hyperplasia of fibrous tissue, with no peritubular capillary vasculitis. Electron microscopy (**Figures 2E, F**) revealed segmental glomerulosclerosis. There were slightly electron-dense homogeneous substances in the segmental mesangial area. Segmental swelling of glomerular capillary endothelial cells and widening of the subendothelial space were noted, with similar deposits in the subendothelial area and numerous red blood cells retained in the capillary loops. Segmental fusion and microvilli formation of foot processes, along with detachment from the basement membrane, were observed. The basement membrane was thickened segmentally, without diagnostically significant stratification in the peritubular capillary basement membrane. Conclusion: Immunofluorescence and electron microscopy showed no significant immunoglobulin or electron-dense deposits. Light microscopy identified glomerular TMA-like lesions, with no evident glomerulitis, tubulitis, or peritubular capillaries or endarteritis. Electron microscopy confirmed segmental fusion of foot processes and absence of stratification in the peritubular capillary basement membrane, indicating no clear rejection reaction. Given the patient’s history, it is likely that drug exposure caused the renal-limited TMA and FSGS-like lesions in the transplanted kidney.

**Figure 3:**
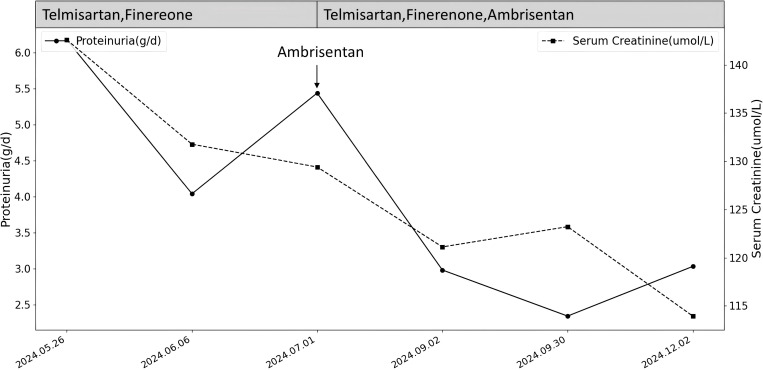
Changes in proteinuria and serum creatinine after stopping the medication and adding RASi and MRA and Ambrisentan for treatment.

Considering the patient’s medical history and renal pathology, bevacizumab was implicated in causing renal-limited TMA with FSGS-like lesions in the transplanted kidney. Following a multidisciplinary consultation with the renal transplantation and oncology departments, it was decided to suspend antitumor drugs and initiate tacrolimus and merti-mecrolide for anti-rejection therapy post-discharge. The patient received full-dose treatment with timosartan, benidipine, fenetylline, and anisentan, resulting in significant reductions in 24-hour urine protein quantification and serum creatinine over 7 months (**Figure 3**). Albumin levels increased from 25g/L to 41g/L. Throughout the 9-month drug discontinuation period, tumor markers and abdominopelvic CT were closely monitored, with no evidence of tumor recurrence or progression.

## Discussion

Post-renal transplantation, patients face a 2–3 fold increased risk of malignant tumors compared to the general population. Key risk factors for the emergence of new tumors following transplantation include being over 40 years of age at the time of the procedure and the long-term use of immunosuppressants postoperatively (9). Statistically, the cumulative incidence of solid organ tumors at 5, 10, and 20 years after renal transplantation is 4-5%, 10%, and greater than 25%, respectively (10). This patient, who underwent renal transplantation at the age of 41, developed small intestinal mucinous carcinoma 17 years later, aligning with the high-risk profile for tumor development.

Bevacizumab, a recombinant humanized anti-VEGF monoclonal antibody, impedes tumor growth by preventing VEGF from binding to its receptor, thereby inhibiting angiogenesis. It has been extensively used in treating various malignancies. In renal tissue, podocytes produce VEGF to protect glomerular endothelial cells from injury. However, bevacizumab disrupts VEGF production by podocytes, directly harming glomerular endothelial cells and compromising the glomerular filtration barrier. Additionally, VEGF pathway inhibition decreases renal endothelial nitric oxide synthase and nitric oxide levels, altering glomerular hemodynamics and worsening proteinuria (2).

Bevacizumab frequently induces proteinuria, though progression to nephrotic syndrome is uncommon. A meta-analysis of 16 clinical trials involving 12,268 patients found a 13.3% incidence of proteinuria, with 2.2% at grade 3 (≥ 3.5 g/d) (6). Reported renal pathologies linked to anti-VEGF agents include thrombotic microangiopathy (TMA), thylakoid proliferative glomerulonephritis, focal segmental glomerulosclerosis, glomerular thylakoid proliferative lesions, and cryoglobulinemic glomerulonephritis. TMA is the most prevalent, marked by endothelial cell swelling, luminal narrowing of small vessels, and microthrombosis. Some patients exhibit renal-limited TMA without the classic triad of thrombocytopenia, microangiopathic hemolytic anemia, and end-organ damage. Biopsy confirms this through features such as double contours, segmental subendothelial expansion, endothelial swelling, and aneurysmal dilatation. An observational study of 100 patients who underwent renal biopsy for hypertension and proteinuria post-anti-VEGF treatment found that 73% exhibited histologic signs of TMA, while 27% showed microscopic nephropathy and collapse-like FSGS (4). Throughout bevacizumab treatment, the patient’s platelet and hemoglobin levels remained stable, with no fever, neurological abnormalities, or signs of systemic TMA. The renal pathology showed endothelial cell swelling, double contours, subendothelial expansion, as well as podocyte proliferation and podocyte foot process fusion, consistent with a mixed renal-limited TMA and FSGS-like lesions pathology. However, the potential contribution of the patient’s renal transplantation history and long-term tacrolimus use as causal or auxiliary factors in the FSGS-like lesions could not be definitively excluded due to the complexity of the case.

During this period, the patient was treated with Sintilimab, a PD-1 inhibitor. Renal damage from PD-1 inhibitors typically results from acute tubulointerstitial nephritis (TIN) (11), with renal-limited TMA being rare. However, in this patient, renal damage persisted despite discontinuing Sintilimab, suggesting these drugs may contribute to renal damage but are not the primary cause. Immunofluorescence revealed lesions characterized by IgA thread-like deposits in capillary collaterals and C3 deposits on the Bowman’s capsule wall. These findings were interpreted as non-significant deposits, likely exudative lesions resulting from endothelial cell injury. This aligns with previous literature indicating that VEGF inhibitor-induced TMA may present with minor immunoglobulin and complement deposits (12). We concluded that the anti-VEGF agent bevacizumab was the primary causative factor in this case.

Recent reports have documented renal damage following renal transplantation associated with bevacizumab use. Müsri et al. described a renal transplant patient with colon cancer who developed severe proteinuria and renal damage post-bevacizumab, with no recovery after cessation of the drug (7).Lawrence Kasherman et al. detailed a case of renal transplantation combined with ovarian cancer where, after bevacizumab administration, there was no progression in proteinuria, only a transient increase in creatinine, which normalized after stopping the drug, with no renal damage upon reapplication of bevacizumab (8). The etiology in these cases remains complex, underscoring the necessity for timely renal biopsy to elucidate the underlying causes. In the aforementioned cases, the absence of renal biopsy left the etiology unresolved. In our case, a rare and comprehensive transplant renal biopsy enabled us to elucidate the renal pathology and analyze the etiology effectively.

There is currently no standard treatment for renal damage induced by anti-VEGF drugs. Literature suggests that most patients with renal-limited TMA achieve partial or complete remission after discontinuing the drug, though some may progress to chronic kidney disease (5, 13, 14). According to the U.S. National Cancer Institute’s Common Terminology Criteria for Adverse Events, treatment should be halted if 24-hour urinary protein exceeds 2 g. Anti-VEGF drugs must be permanently discontinued if nephrotic syndrome or TMA develops (15). Oncologists must assess tumor stability before withdrawal, as tumor recurrence following anti-VEGF discontinuation is frequently reported (16).

Four years post-tumor discovery, despite stage IV classification with widespread metastases and over 40 chemotherapy cycles, the patient’s left ileal wall thickening and multiple pelvic masses have resolved, leaving only a reduced low-density shadow in the right pelvis, shrinking from 6.0×4.4 cm to 1.8×1.5 cm. After thorough consultation with oncologists, the tumor was deemed relatively stable. However, the patient’s renal symptoms had significantly worsened, prompting the decision to cease drug treatment.

Renin-angiotensin-aldosterone system inhibitors (RASi) are recommended for non-specific kidney treatment as they lower glomerular perfusion pressure and reduce urinary protein excretion (17). Renal-limited TMA often involves podocyte lesions, with proteinuria correlating with foot process effacement (18). In this case, podocyte hyperplasia and foot process fusion were noted. Consequently, finerenone, known for its podocyte-protective effects, was selected. Despite discontinuing anti-tumor drugs and administering telmisartan and finerenone for a month, the 24-hour urinary protein quantification showed minimal change, decreasing from 6.18 g/d to 5.44 g/d. Given that endothelial cell damage is the primary pathological mechanism of renal-limited TMA, we introduced ambrisentan, an endothelin receptor antagonist (ERA).

Endothelin-1 (ET-1), a potent endogenous vasoconstrictor, exerts significant pathophysiological effects on the kidney via endothelin receptor A (ETA), causing podocyte and mesangial cell injury, increased glomerular pressure from efferent arteriole constriction, and tubulointerstitial fibrosis due to inflammation and immune activation (19). Ambrisentan, a selective ETA antagonist, mitigates ET-1’s effects by protecting endothelial cells, dilating glomerular vessels, reducing filtration pressure, and decreasing urinary protein, thus offering renal protection (20). Recent clinical trials, including the PROTECT (21)and SONAR (22) studies, have confirmed the efficacy of endothelin receptor antagonists in reducing proteinuria in chronic kidney disease patients, demonstrating good safety and tolerability. In this case, ambrisentan treatment for two months led to a marked reduction in 24-hour urinary protein from 5.44 g to 2.98 g, maintaining stability between 2 and 3 g over the subsequent five months.

In conclusion, the etiology of kidney damage in post-transplant patients with tumor complications is intricate, necessitating early renal biopsy for definitive diagnosis. We present the inaugural case of a post-kidney transplantation patient with a malignant tumor undergoing renal biopsy, which revealed nephrotic syndrome due to renal-limited TMA with FSGS-like lesions. The patient’s renal pathology was notably complex and diverse. Due to incomplete pre-transplant history and missing donor information, our attempts at a differential diagnosis could not fully elucidate all pathological findings. Considering the pronounced features of renal-localized TMA, we identify bevacizumab as a primary etiological factor for the nephrotic syndrome. Following the cessation of anti-tumor medication and the implementation of non-specific treatment, a significant reduction in proteinuria was observed. No signs of tumor progression were observed, but long-term prognosis still requires close follow-up. This case underscores the critical role of multidisciplinary evaluation in refining treatment strategies. Currently, beyond the discontinuation of suspected drugs, there are no established diagnostic and treatment guidelines for such scenarios. Non-specific therapeutic agents such as RAS inhibitors and MRAs continue to be vital treatment options. Although this patient showed notable improvement with ERA, further clinical data are needed to substantiate its long-term efficacy.

## Data Availability

The original contributions presented in the study are included in the article/supplementary material. Further inquiries can be directed to the corresponding author.
